# Internet use and social trust: empirical analysis based on CGSS2021

**DOI:** 10.3389/fsoc.2024.1422731

**Published:** 2025-01-03

**Authors:** Juan Miao, Junfeng Kuang, Linlin Yang, Ming Chen, Xueqing Tian

**Affiliations:** ^1^School of Law and Political Science, Yunnan University of Finance and Economics, Kunming, China; ^2^Southwest Frontier Minority Research Center, Yunnan University, Kunming, China; ^3^College of Humanities and Social Science, Yunnan Agricultural University, Kunming, China

**Keywords:** Internet use, social trust, perception of social fairness, social support, social interaction

## Abstract

The development of the Internet has significantly changed the way people live and interact with each other. Interaction is the foundation for building trust and may therefore also be influenced by the Internet. This study aims to examine the impact of Internet use on different dimensions of social trust, focusing on the roles of perceived fairness and social support, using the latest data from the CGSS from China. The results show that Internet use has a significant negative predictive effect on the level of social trust, and the perception of social fairness plays a fully mediating role in this relationship. That is, Internet use can indirectly reduce people’s level of social trust by reducing their perception of social fairness. Furthermore, the results indicate that social support can moderate people’s perceptions of social fairness and thus mitigate the negative effects of Internet use on social trust. These results suggest that we should raise the profile of the impact of internet use, actively improve people’s perceptions of social fairness to increase their level of social trust, and finally, focus on the positive role of social support, which can reduce the negative impact of internet use.

## 1 Introduction

Trust is the lubricant for social and economic transactions and is also considered the cornerstone of behavioural economic research (Camerer, 2003; Ermisch et al., 2009). Trust levels act as an indicator and a proxy variable (Leigh, 2006), and high levels of trust are usually associated with higher economic growth and financial development (Knack and Keefer, 1997; Guiso et al., 2004; Bjørnskov, 2017). Trust can be divided into particular trust and general trust (Steinhardt and Delhey, 2020). This paper focuses on social trust (general trust), which usually refers to trust in strangers or people in general (Durlauf and Fafchamps, 2005). The level of social trust people had can affect their judgements about the behaviour of others, which in turn affects their willingness to cooperate with others (Mell et al., 2022). Previous research has found that age, gender, socio-economic status, social time, and childhood experiences may have an impact on social trust (Patulny, 2011; Azzollini, 2023; Yang et al., 2023), but there is still less research on the factors influencing social trust. Hence, we need to identify the factors influencing social trust and work towards increasing people’s level of social trust, which is important for promoting cooperation and economic development.

As China’s internet infrastructure continues to improve, the country’s internet users are also growing rapidly. CNNIC (China Internet Network Information Centre) released the 51st “Statistical Report on China’s Internet Development” on March 2 (CNNIC, 2023).^1^ The report shows that until December 2022, the number of Internet users in China was 1.067 billion, and the Internet penetration rate reached 75.6%. Internet use has greatly changed the way people live and interact with each other (Nie et al., 2017). And as interaction is considered key to building trust, changes in the form of interaction may also have a significant impact on trust (Liu, 2023). While some researchers have found that Internet use does affect social trust, the size and direction of the effect remain controversial (Wang and Zhou, 2019). Therefore, more empirical research is necessary to fully understand the impact of Internet use on social trust and the mechanisms that influence it.

The perception of social fairness is considered to be one of the key influences on trust. Fairness is the foundation of trust, and trust arises when social fairness is felt (Brugman et al., 2016). People who live in a fair world may have more confidence in the world, and confidence will be reflected in trust in others (Wang et al., 2020). Hence, people’s perception of social fairness may also affect their social trust. In addition, the Internet affects people’s interactions (Putnam, 2000), and interactions with family, friends, etc., are seen as part of social support (Lai et al., 2023). Social support can alleviate people’s stress and change their attitudes toward society (Kalaitzaki et al., 2021), which may, in turn, affect their perception of social fairness. Therefore, investigating the mechanisms of the impact of Internet use on social trust by studying social support and the perception of social fairness will be helpful.

**Figure 1:**
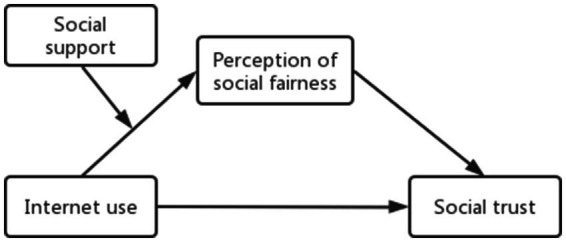
Research framework and hypotheses.

This paper uses the latest data to analyse the relationship between Internet use and social trust. We draw a research model framework (see Figure 1). Then, we will check the effect of the perception of social fairness and social support in this relationship. We hope the results of this study contribute to research on social trust.

## 2 Literature review and hypothesis

### 2.1 Internet use and social trust

Internet use in this paper refers to the frequency of an individual’s use of the Internet, which reflects the importance of the Internet as a source of information. Information is the foundation of people’s choices and judgements and is a key factor in forming perceptions. The cultivation theory was first proposed by Gerbner, who found that people’s use of the television medium influenced their behaviour and cognition (Gerbner, 1969). Other scholars have since extended their research from the television medium to other mediums, arguing that the messages in the medium can subtly change people’s behaviour and cognition as they use it (Lu and Wei, 2023). Trust, as an expectation and aspiration, is also a judgement of people based on the information they currently have (Liu, 2023; Barber, 1983). Therefore, people are more likely to rely on the information to make choices and judgements when they go online more frequently, so the information on the Internet may have a greater impact on them. In addition, current research has shown that Internet use has negative effects on social trust in different groups, such as young people and older people (Lu and Wei, 2023; Zhao and Li, 2017; Sabatini and Sarracino, 2019).

There are three probable reasons for this. First, the interaction on the Internet is virtual and anonymous. While the Internet is conducive to building interactions, it may also weaken the intensity of the interactions. The virtual and anonymous characters of the Internet can make it more difficult to predict the behaviour of the interacting parties, which is not helpful in monitoring and regulating the behaviour of both parties (Wang and Emurian, 2005). Therefore, trust in the Internet will face greater risks (Guan, 2015).

Second, Internet use has a time-substitution effect. Putnam found that television privatises leisure time and reduces the time and desire to engage in social activities, which are necessary for social trust to emerge, and that reduced social engagement is detrimental to social trust building (Putnam, 2000). The Internet is more private than television, and as an entertainment pastime, it competes with face-to-face social time, which may further reduce people’s social participation (Nie et al., 2002).

Third, people’s information selection preferences and exposure to conflicting events. On the one hand, people have a negative preference for the choice of information (Vaish et al., 2008), and people tend to focus on dangerous and negative information to survive (Zhao and Li, 2017). The Internet has less regulation and more negative information than traditional media, and the Internet has also greatly increased individual autonomy over information choices, so people may be exposed to more negative information. Misinformation and the mutual negativity of negative information can have an impact on reducing people’s trust (Zhao and Wang, 2021). On the other hand, the Internet makes it easy for people to be exposed to conflicting events, but long-term exposure to conflicting events may cause people to lower their social trust to protect themselves (Lewis and Topal, 2023). In combination, therefore, we propose hypothesis 1.

**Hypothesis 1**: Internet use has a negative effect on social trust.

### 2.2 The mediating effect of the perception of social fairness

The perception of social fairness refers to people’s subjective evaluations of the current state of fairness in their interactions (Xie et al., 2022). People’s perceptions of how they have been unfairly treated can affect their beliefs, attitudes, and behaviors (Brockner and Wiesenfeld, 1996) and may also have a significant impact on their subjective well-being (Sun and Xiao, 2012; Ugur, 2021). According to social exchange theory, people use trust as compensation for fairness in interactions with others, and unfair treatment can erode people’s trust in others (Cheung et al., 2022). Current research has shown that the perception of social fairness will affect trust, and individuals’ perceptions of social fairness are significantly related to social trust (Cheung et al., 2022; You, 2012; Sun et al., 2021; Li and He, 2022). Valcke et al. used an experiment to study social belonging among African and Hispanic Americans and found that minority members’ sense of social belonging grows when perceived procedural fairness increases, which in turn increases their social trust (Valcke et al., 2020).

People’s perception of social fairness is influenced not only by experience but also by observation. Research by Yuan Bo et al. has further shown that both perceived and observed fairness affect people’s social trust (Yuan et al., 2023). People’s sources of information were expanded by the Internet, so when people use the Internet more frequently, on the one hand, they may observe more incidents of injustice, which leads to a decrease in people’s perception of social fairness; on the other hand, the Internet broadens people’s reference objects, which will increase people’s sense of relative deprivation when the upward comparison is triggered, thus leading to a decrease in their sense of social justice (Xie et al., 2022). Studies have shown that there is a significant correlation between Internet use and perceptions of social justice (Xie et al., 2022; Zhu et al., 2020; Zhou et al., 2022). In terms of impact mechanisms, researchers have found that Internet use may lead to a decline in the perception of social fairness by reducing people’s sense of income equity and class mobility (Li et al., 2023) and may also lead to a reduced perception of social fairness by increasing individuals’ tendency to make outward-looking attributions (Han and Pan, 2023). Combining the above studies, we propose Hypothesis 2.

**Hypothesis 2**: The perception of social fairness plays a mediating role in the relationship between Internet use and social trust.

### 2.3 The moderating effect of social support

Social support refers to resources and help that individuals can receive from others, which can enhance their social adjustment at both the spiritual and material levels (Cohen et al., 1985). Cohen and Wills found that social support has a gain effect and a buffer effect, which can buffer individuals from stress when coping with negative events (Cohen and Wills, 1985). Caplan argues that social support comes from the social network in which they are embedded and is provided through connections within that social network (Caplan, 1974). Therefore, it can be considered that close ties with people such as family, friends, colleagues, and neighbors contribute to the formation of good social networks, which can not only provide us with emotional support and relieve stress but also provide material help to cope with difficulties.

In emotional terms, social support facilitates the alleviation of perceived stress and improves mental health (Cohen and Wills, 1985; Szkody and McKinney, 2019). As well, perceived stress may have an impact on the perception of social fairness (Ma and Ma, 2019; Xu et al., 2021; Zhang and Zhu, 2022). Thus, social support may contribute to reducing perceptions of inequity by alleviating stress. In material terms, better social interaction helps to maintain social networks. And social networks, as a form of social capital (Stern and Putnam, 1993), can help us overcome difficulties. Frustrations and difficulties experienced are important factors in the creation of a sense of unfairness (Berkowitz, 1972; Luo and Wei, 2009; Zhu, 2018). Zhu et al. also showed that social capital, with social networks as a proxy variable, had a significant positive impact on residents’ perceptions of fairness (Zhu et al., 2021).

Internet use has a negative effect on people’s perceptions of fairness (Xie et al., 2022), and social support can mitigate perceptions of unfairness (Zhu et al., 2021). Therefore, when more social support exists, this may help mitigate the negative effects of Internet use on the perception of social fairness. Based on the effects of Internet use and social support on the perception of social fairness, we propose Hypothesis 3.

**Hypothesis 3**: Social support plays a moderated role in the relationship between Internet use and perception of social fairness.

Based on the above research and hypotheses, we suggest that social support may moderate the mediating role of the perception of social fairness between Internet use and social trust. Social support mitigates perceived unfairness, and therefore, when Internet use affects social trust by influencing the perception of social fairness, it may also be influenced by social support. In other words, when the level of social support is higher, the effect of Internet use on social trust through the perception of social fairness will be weaker. Therefore, we propose Hypothesis 4.

**Hypothesis 4**: Social support moderates the mediating effect of the perception of social fairness between Internet use and social trust.

## 3 Methods

### 3.1 Data and sample

The data used in this study comes from CGSS 2021 (Chinese General Social Survey). The Chinese General Social Survey is China’s earliest national, comprehensive, and continuous academic survey project, which started in 2003. The survey data of this project covered information of Chinese social members in various aspects, including economy, education, and lifestyle, providing rich data support for the exploration of the relationship between Internet use and social trust. Data is available at: http://www.cnsda.org/. CGSS 2021 is the latest publicly available survey we can use. The total number of samples involved in the CGSS2021 database was 8,148. After dealing with the missing values in the data, there were 6,220 valid samples remaining.

### 3.2 Measures

#### 3.2.1 Dependent variable

In this study, the dependent variable is social trust. Drawing on the experience of previous scholars (Deng and Yu, 2021), we measured the variable of social trust by using the question “Generally speaking, do you agree that almost everyone in society is trustworthy?” in the CGSS2021 database. The scores for this question were calculated using a 5-point Likert scale, where 1 represents “strongly disagree” and 5 represents “strongly agree.”

#### 3.2.2 Independent variable

In this study, the independent variable is Internet use. We measured the variable of Internet use by using the question “How often did you use the Internet (including mobile Internet) in the past year?” in the CGSS2021 database. The scores for this question were calculated using a 5-point Likert scale, with 1 indicating “never” and 5 indicating “very frequently.”

#### 3.2.3 Mediating variable

In this study, the mediating variable is the perception of social fairness. Although there are different dimensions of the perception of social fairness, the overall perception of social fairness also plays an important role in people’s behavior (Zhou et al., 2016). Therefore, we measured the variable of the perception of social fairness by using the same question (“Overall, do you think today’s society is fair or unfair?”) in CGSS2021 (Xie et al., 2022). The scores for this question were calculated using a 5-point Likert scale, with 1 indicating “completely unfair” and 5 indicating “completely fair.”

#### 3.2.4 Moderating variable

In this study, the moderating variable is social support. Drawing on previous research (Zhu et al., 2021), we use the frequency of social and recreational activities that individuals engage in with neighbors and friends as a proxy variable for social support. The measurement of the variable of social support involves two questions in the CGSS2021 database: “How often do you participate in social and recreational activities with neighbors or other friends (for example, visiting each other’s homes, watching TV, having meals together, playing cards, etc.)?” Firstly, we converted the answers into a 3-point Likert scale, where 0 represents “never,” 1 represents “once a month or less,” and 2 represents “more than once a month.” Then, the scores of the two questions were averaged.

#### 3.2.5 Control variables

Drawing on previous research and taking into account the influencing factors of social trust (Awaworyi Churchill and Mishra, 2017; Li and Jiang, 2022), we selected gender (female = 0, male = 1), age (survey time minus birth year), educational level (primary school and below = 0, junior middle school = 1, high school = 2, college above = 3), marital status (unmarried = 0, married = 1), household registration (agricultural residence = 0, non-agricultural residence = 1), health status (very unhealthy = 1, very good health = 5), and political status (non-party member = 0, party member = 1) as control variables in this study.

### 3.3 Data analysis

The data analysis in this investigation followed a comprehensive three-step analytical protocol. Firstly, we employed SPSS 25.0 to conduct descriptive statistics and correlation analysis on the research data. Subsequently, we tested the regression model, moderating effects, and mediating effects by running SPSS 25.0. Finally, a robustness test was conducted on the research results.

## 4 Results

### 4.1 Descriptive statistics and correlations

The descriptive results (Table 1) indicate that in terms of age, the average age of the samples is 51.94 years, with a standard deviation of 16.754 years. Regarding gender, approximately 46% are male and 54% are female. In respect of marital status, the majority of the respondents are married (86%), including those who are divorced, widowed, or remarried. According to the household registration, 59% are from agricultural households. Moreover, the mean value of social trust is 3.67 (SD = 0.989), and the mean value of Internet use is 3.37 (SD = 1.651). Meanwhile, the mean value of the perception of social fairness is 3.46 (SD = 0.971), and the mean value of the perception of social support is 1.22 (SD = 0.653).

**Table 1:** Descriptive statistics.

Mean(SD)							
Variables	Male	Female	Rural	Urban	Total (SD)	Min	Max
Social trust	3.73 (0.98)	3.61 (0.99)	3.67 (1.01)	3.67 (0.95)	3.67 (0.989)	1	5
Internet use	3.35 (1.67)	3.38 (1.64)	3.08 (1.68)	3.78 (1.52)	3.37 (1.651)	1	5
Perception of fairness	3.54 (0.95)	3.39 (0.98)	3.46 (1.01)	3.47 (0.91)	3.46 (0.971)	1	5
Social support	1.22 (0.64)	1.21 (0.66)	1.23 (0.67)	1.20 (0.63)	1.22 (0.653)	0	2
Age	52.74 (17.02)	51.25 (16.50)	52.05 (16.10)	51.79 (17.64)	51.94 (16.754)	18	94
Gender			0.45(0.50)	0.48(0.50)	0.46(0.499)	0	1
Income	10.45 (2.44)	10.36 (2.48)	9.89 (2.70)	11.13 (1.84)	10.40 (2.462)	0	16.12
Education	1.36 (1.09)	1.18 (1.13)	0.88 (0.97)	1.81 (1.07)	1.26 (1.112)	0	3
Marital status	0.84 (0.37)	0.88 (0.32)	0.88 (0.33)	0.84 (0.37)	0.86 (0.345)	0	1
Household registration	0.42 (0.49)	0.40 (1.10)			0.41 (0.492)	0	1
Health	3.52 (1.07)	3.43 (1.10)	3.40 (1.15)	3.56 (0.99)	3.47 (1.087)	1	5
Political status	0.19 (0.39)	0.08 (0.19)	0.07 (0.25)	0.22 (0.41)	0.13 (0.335)	0	1

Table 2 presents the correlation results among the various research variables. The perception of social fairness (r = 0.322, *p* < 0.01) and social support (r = 0.039, *p* < 0.01) show a significantly positive correlation with social trust, while Internet use has a significantly negative correlation with social trust (r = −0.088, *p* < 0.01). Furthermore, Internet use shows a significantly positive correlation with social support (r = 0.155, *p* < 0.01) but has a significantly negative correlation with the perception of social fairness (r = −0.081, *p* < 0.01). Finally, social support has a significantly positive correlation with the perception of social fairness (r = 0.033, *p* < 0.01).

**Table 2:** Correlations among variables.

	1	2	3	4	5	6	7	8	9	10	11	12
1	1											
2	−0.088**	1										
3	0.322**	−0.081**	1									
4	0.039**	0.155**	0.033**	1								
5	0.133**	−0.611**	0.098**	−0.138**	1							
6	0.060**	−0.010	0.075**	0.014	0.044**	1						
7	−0.003	0.308**	0.009	0.032*	−0.268**	0.017	1					
8	−0.018	0.514**	−0.006	0.086**	−0.513**	0.080**	0.336**	1				
9	0.031*	−0.221**	0.026*	−0.061**	0.459**	−0.066**	−0.089**	−0.320**	1			
10	−0.001	0.207**	0.004	−0.021	−0.008	0.023	0.248**	0.415**	−0.052**	1		
11	0.019	0.321**	0.081**	0.152**	−0.356**	0.040**	0.202**	0.279**	−0.165**	0.072**	1	
12	0.080**	0.065**	0.073**	−0.003	0.103**	0.159**	0.110**	0.248**	0.047**	0.225**	0.053**	1

1: Social trust, 2: Internet use, 3: Perception of fairness, 4: Social support, 5: Age, 6: Gender, 7: Income, 8: Education, 9: Marital status, 10: Household registration, 11: Health, 12: Political status.

*p < 0.05 (2-tailed), **p < 0.01 (2-tailed).

### 4.2 Test of regression model

Five models are used to analyze if Internet use affected the perception of social fairness, social support, and social trust. According to the results in Table 3, all hypotheses were supported.

**Table 3:** Regression coefficient and significance of the model.

	Model 1 (Outcomes: ST)	Model 2 (Outcomes: POF)	Model 3 (Outcomes: POF)	Model 4 (Outcomes: ST)	Model 5 (Outcomes: ST)
	B (β)	B (β)	B (β)	B (β)	B [Exp (B)]
Age	0.010 (0.173)***	0.007(0.125)***	0.007(0.128)***	0.008(0.137)***	0.015(1.015)***
Gender	0.071 (0.036)**	0.103 (0.053)***	0.100 (0.051)***	0.038 (0.019)	−0.021 (0.979)
Income	0.010 (0.025)	0.010 (0.026)	0.010 (0.025)	0.007 (0.018)	0.038 (1.039)*
Education	0.052 (0.058)**	0.040 (0.046)*	0.041 (0.047)**	0.039 (0.044)*	0.090 (1.095)
Marital status	−0.079 (−0.028)	−0.026 (−0.009)	−0.022 (−0.008)	−0.070 (−0.024)	−0.192(0.825)
Household registration	−0.080 (−0.040)**	−0.046 (−0.023)	−0.045 (−0.023)	−0.060 (−0.030)*	0.014 (1.041)
Health	0.061 (0.067)***	0.110 (0.123)***	0.109 (0.122)***	0.022 (0.025)	0.040 (1.041)
Political status	0.144 (0.049)***	0.121 (0.042)**	0.123 (0.042)**	0.107 (0.036)**	0.236 (01.266)
Internet use	−0.025 (−0.042)*	−0.047 (−0.081)***	−0.101 (−0.172)***	−0.014 (−0.024)	−0.022 (0.978)
Perception of fairness				0.305 (0.300)***	0.649 (1.914)***
Social support		0.055 (0.037)**	−0.087 (−0.058)*	0.063 (0.042)**	0.204 (1.227)***
Internet use*Social support			0.047 (0.153)***		
R^2^	0.032	0.037	0.039	0.122	0.071

ST, Social trust; POF, Perception of fairness.

*p < 0.05, **p < 0.01, ***p < 0.001.

Model 1 showed that, with control variables added in, Internet use had a negative effect on social trust (β = −0.042, *p* < 0.05). People who use the Internet more frequently were more likely to have lower social trust. Hypothesis 1 was supported.

In Model 2 and Model 3, the perception of social fairness is the outcome. Model 2 suggested that both Internet use (β = −0.081, *p* < 0.001) and social support (β = 0.037, *p* < 0.01) had a significant effect on the perception of social fairness. Model 3 indicated the product of Internet use and social support had a positive effect on the perception of social fairness. Combined with Model 2 and Model 3, hypothesis 3 was supported; that is, social support may play a moderating role between Internet use and perceived social fairness.

In Model 4 with social trust as the outcome, the result showed that the effect of Internet use on social trust is no longer significant when the perception of social fairness was included in the model (β = −0.024, *p* > 0.05). And the perception of social fairness had a significant positive effect on social trust; that is, people who have a high level of perception of social fairness were likely to have a higher social trust. The above results of Model 1 and Model 4 indicated that the perception of social fairness may play a mediating role between Internet use and social trust. That is, Internet use indirectly influenced people’s social trust through the perception of social fairness. Therefore, hypothesis 2 was supported.

### 4.3 Test of mediation effect and moderated mediation effect

Table 4 presented the results of mediating and moderating effects. In the mediation model, the total effect (Effect = −0.0253, SE = 0.0102, 95% CI = [−0.0452, −0.0054]) and indirect effect (Effect = −0.0138, SE = 0.0032, 95% CI = [−0.0201, −0.0075]) were significant; the bootstrap CI (95%) in both paths did not include zero. But the direct effect was not significant (Effect = −0.0115, SE = 0.0097, 95% CI = [−0.0305, 0.0075]), and the bootstrap CI (95%) includes zero in this path. The result showed that the perception of social fairness plays a fully mediated role between Internet use and social trust. This mediation model was supported. Therefore, hypothesis 2 was verified once again.

**Table 4:** Mediation and moderated mediation effect.

Mediation effect	Effect	BootSE	BootLLCI-95%	BootULCI-95%
Total effect	−0.0253	0.0102	−0.0452	−0.0054
Direct effect	−0.0115	0.0097	−0.0305	0.0075
Indirect effect	−0.0138	0.0032	−0.0205	−0.0075
Moderated mediation effect	Effect	BootSE	BootLLCI	BootULCI
Low (−1SD)	−0.0230	0.0041	−0.0312	−0.0151
Mean	−0.0137	0.0033	−0.0203	−0.0073
High (+1SD)	−0.0043	0.0040	−0.0122	0.0033
Index of moderated mediation	0.0143	0.0035	0.0074	0.0212

In addition, the result of the moderated mediation effect showed that social support had a moderated mediation effect in the model. The indirect effect was evaluated at 1 SD below and above the mean shown in Table 4. The mediation effect of the perception of social fairness between Internet use and social trust became attenuated at 1 SD below the mean of social support (Effect = −0.0230, SE = 0.0041, 95% CI = [−0.0312, −0.0151]). Hypothesis 4 was supported.

### 4.4 Robust test

To further test the robustness of the study results, this paper converts social trust into a 2-point variable, with 1 and 2 converted to 0, indicating distrust, and 3, 4, and 5 converted to 1, indicating trust. Internet use, sense of social fairness, social trust, and the control variables were added to the regression model, and the results are shown in Model 5. The results are basically the same as before, except for the control variables of household status and political outlook, which became insignificant, so the study results can be considered robust.

## 5 Conclusion and discussion

Based on the latest survey data from CGSS, we investigate the relationship between Internet use and social trust. The results of this study have shown that three main hypotheses were supported. The findings support the notion that Internet use negatively affects social trust and can indirectly impact social trust by weakening the perception of social fairness. This study also found that social support plays a moderate role. Social support can moderate the mediating effect of the perception of social fairness between Internet use and social trust.

First, our research shows that Internet use significantly reduces people’s social trust, which is consistent with previous studies (Lu and Wei, 2023; Sabatini and Sarracino, 2019). With the rapid development of the economy, the Internet has become integrated into the lives of ordinary people. However, due to the lack of effective regulation, there is a huge amount of negative and misleading information on the internet, which can have a subtle effect on people’s perceptions (Gerbner, 1969). In addition, the current Internet media is driven by profit and is keen to stir up confrontation and conflict. Exposure to conflicting incidents on the Internet may also reduce people’s social trust (Lewis and Topal, 2023). The above may be the reasons for the reduction in people’s social trust. Therefore, better management of negative information on the Internet is necessary.

Second, Internet use can affect social trust by influencing people’s perceptions of fairness. This is in keeping with previous research findings (Zhu et al., 2020). This study shows that Internet use does affect attitudes and perceptions. The more frequently the Internet is used, the more likely people are to have a lower perception of social fairness, which leads to a lower level of social trust. The possible reason for this is that the Internet expands people’s objects of reference, and the expansion of upward comparisons increases people’s sense of relative deprivation (Xie et al., 2022). At the same time, the Internet provides more information and facilitates social interaction, increasing the unfairness people observe and the probability of experiencing it (Zhou et al., 2022). These may be the reasons why internet use reduces the perception of social fairness. It also provides a new explanation for the impact of Internet use on social trust.

Third, the results of this study show that social support has a significant positive impact on the perception of social fairness, which is consistent with previous research (Zhu et al., 2021). In addition, social support has a moderating effect, which can mitigate the negative effects of Internet use on the perception of social fairness. Social support can also moderate the mediating role of the perception of social fairness between Internet use and social trust. One of the reasons may be that social support helps to alleviate negative emotions and stress, and can also have an impact on people’s perceptions and attitudes (Cohen and Wills, 1985). Thus, when higher levels of social support exist, people can reduce the negative impact of internet use on their perception of social fairness, which can also affect the whole mediation model. That means a higher level of social support can reduce the negative effect of Internet use on social trust by enhancing the perception of social fairness. The findings on social support have significant implications for the study of Internet use.

## 6 Practical implications

Based on the above research, there are some targeted policy suggestions for enhancing the level of social trust. First, Government needs to focus on the impact of Internet use. On the one hand, there is a need to limit and reduce the dissemination of false and erroneous information and to decrease the impact of negative information on people’s perceptions. On the other hand, effective regulation needs to be provided for transactional behavior on the Internet to enhance people’s social trust at an institutional level.

Second, this study shows that the perception of social fairness was related to social trust, and the perception of social fairness plays a mediating role between Internet use and social trust. Thus, the government should work to improve people’s perception of social fairness. Improving the social security system and narrowing the gap between the rich and the poor play an important role in reducing the sense of relative deprivation, which contributes to a greater sense of social fairness.

Finally, due to the moderating effect of social support, the government should promote higher levels of social support and encourage people to interact with family and friends offline. Maintaining a strong network is not only good for overcoming difficulties but also provides spiritual comfort, which can improve people’s attitudes and cognition. When perceived social support improves, people will have a higher perception of social fairness, which can also increase social trust.

## 7 Limitations and future research

As is the case with any academic exploration, this study inevitably has its limitations, and these aspects call for meticulous attention in subsequent research undertakings. Firstly, there is a problem with data timeliness that the survey time was 3 years ago, although we have used the most recent publicly available data. So, it is necessary to collect first-hand data in future research. Secondly, we use cross-sectional data rather than longitudinal data. But trust changes as society evolves, and cross-sectional data does not reflect this change. Therefore, we should use longitudinal data to test the extent of change in the social trust of the Chinese people and the causal relationship with Internet use in further research. Thirdly, while some scholars believe that self-reported levels of trust have some reference value, people’s trust behaviors do not necessarily correspond to self-reported levels of trust. Therefore, several dimensions are needed in future research to determine people’s true SOCIAL trust levels.

## Data Availability

The original contributions presented in the study are included in the article/supplementary material, further inquiries can be directed to the corresponding author.

## Footnotes

^1^ https://www.cnnic.net.cn/n4/2023/0303/c88-10757.html
